# Creation of a Laboratory for Statistics and Analysis of Dependence and Chronic Conditions: Protocol for the Bages Territorial Specialization and Competitiveness Project (PECT BAGESS)

**DOI:** 10.2196/46542

**Published:** 2023-07-26

**Authors:** Georgina Pujolar-Díaz, Josep Vidal-Alaball, Anna Forcada, Elisabet Descals-Singla, Josep Basora, PECT BAGESS Group

**Affiliations:** 1 Unitat de Suport a la Recerca de la Catalunya Central Fundació Institut Universitari per a la Recerca a l'Atenció Primària de Salut Jordi Gol i Gurina Sant Fruitós de Bages Spain; 2 Health Promotion in Rural Areas Research Group Gerència Territorial de la Catalunya Central Institut Català de la Salut Sant Fruitós de Bages Spain; 3 Faculty of Medicine University of Vic-Central University of Catalonia Vic Spain; 4 Gerència Territorial de la Catalunya Central Institut Català de la Salut Sant Fruitós de Bages Spain; 5 Servei d’Atenció Primària Bages-Berguedà-Moianès Gerència Territorial de la Catalunya Central Institut Català de la Salut Sant Fruitós de Bages Spain; 6 Fundació Institut Universitari per a la Recerca a l'Atenció Primària de Salut Jordi Gol i Gurina Barcelona Spain; 7 Grup de Recerca en Innovació Transformativa i Simulació Unitat de Innovació i Recerca de Manresa University of Vic-Central University of Catalonia Manresa Spain; 8 Unitat de Recerca i Innovació Althaia Xarxa Assistencial Universitària de Manresa – Fundació Privada Manresa Spain; 9 Unitat de Recerca i Innovació Sant Andreu Salut Manresa Spain; 10 Eurecat Centre Tecnològic Manresa Spain; 11 Fundació Ampans Manresa Spain; 12 Servei d’Alcaldia i Presidència Ajuntament de Manresa Manresa Spain

**Keywords:** chronic disease, multiple chronic conditions, primary health care, diffusion of innovation, health data, data sharing

## Abstract

**Background:**

With the increasing prevalence of chronic diseases, partly due to the increase in life expectancy and the aging of the population, the complexity of the approach faced by the structures, dynamics, and actors that are part of the current care and attention systems is evident. The territory of Bages (Catalonia, Spain) presents characteristics of a highly complex ecosystem where there is a need to develop new, more dynamic structures for the various actors in the health and social systems, aimed at incorporating new actors in the technological and business field that would allow innovation in the management of this context. Within the framework of the Bages Territorial Specialization and Competitiveness Project (PECT BAGESS), the aim is to address these challenges through various entities that will develop 7 interrelated operations. Of these, the operation of the IDIAP Jordi Gol-Catalan Health Institute focuses on the creation of a Laboratory for Statistics and Analysis of Dependence and Chronic Conditions in the Bages region, in the form of a database that will collect the most relevant information from the different environments that affect the management of chronic conditions and dependence: health, social, economic, and environment.

**Objective:**

This study aims to create a laboratory for statistical, dependence, and chronic condition analysis in the Bages region, to determine the chronic conditions and conditions that generate dependence in the Bages area, in order to propose products and services that respond to the needs of people in these situations.

**Methods:**

PECT BAGESS originated from the Shared Agenda initiative, which was established in the Bages region with the goal of enhancing the quality of life and fostering social inclusion for individuals with chronic diseases. This study presents part of this broader project, consisting of the creation of a database. Data from chronic conditions and dependence service providers will be combined, using a unique identifier for the different sources of information. A thorough legal analysis was conducted to establish a secure data sharing mechanism among the entities participating in the project.

**Results:**

The laboratory will be a key piece in the structure generated in the environment of the PECT BAGESS, which will allow relevant information to be passed on from the different sectors involved to respond to the needs of people with chronic conditions and dependence, as well as to generate opportunities for products and services.

**Conclusions:**

The emerging organizational dynamics and structures are expected to demonstrate a health and social management model that may have a remarkable impact on these sectors. Products and services developed may be very useful for generating synergies and facilitating the living conditions of people who can benefit from all these services. However, secure data sharing circuits must be considered.

**International Registered Report Identifier (IRRID):**

PRR1-10.2196/46542

## Introduction

The prevalence of chronic diseases has increased worldwide over recent decades and is no longer a characteristic of developed countries [1,2]. These types of diseases tend to be long-lasting, generally slow to progress, and noncommunicable among people [1,3,4]. Life expectancy has increased as a result of social progress and advances in health [4]. However, this has led to an increased risk of chronic multimorbidity, especially among older adults [2,4,5].

As a result, an increase in the burden of noncommunicable and chronic diseases is being described globally [1,4,6,7]. Consequently, there is an increase in years lived with disability and eventually in situations of dependence. Altogether, this poses a challenge from multiple perspectives. Health systems, and especially primary care, must adopt a complex approach in terms of detection, care, and follow-up of cases of chronic conditions or chronic multimorbidity, in addition to the costs involved [2,4,8]. There is also a direct impact on the loss of quality of life of people with these conditions and the emerging needs they develop [2,4,5]. At the same time, the circumstances of chronic conditions increase the fragmentation and burden of care, as well as the burden on family relationships and carers [5,9-13].

The difficulty of dealing with chronic diseases often lies in the complexity of the structures, dynamics, and actors involved in care, beyond the clinical complexity that care for these conditions may entail. Given the diversity of chronic diseases and their impact on the people who have them, management that takes into account a multiplicity of factors is necessary. This requires patient identification, comprehensive needs assessments, management of needs and resources, coordination between health and social agents that can influence an optimal care response, ensuring round-the-clock care, minimizing potentially avoidable hospitalizations, and accompanying people at the end of their life trajectory when appropriate [4].

The Catalan health system (integrated within the Spanish national health system) is intended to provide universal care and is financed by citizens’ taxes, based on a public model centered on personal care and a comprehensive approach to health [14]. In this context, primary care plays an important role in the clinical management of chronic diseases and cases of patients with complex conditions. Thus, the system allows for continuity and longitudinality of care, together with other levels of care (emergency, specialist consultations, and hospital admissions) [4,15]. In parallel, sociohealth and social structures provide care focused on aspects derived from clinical conditions (eg, home care and formal care) [10]. Together, the various health and social agents make it possible to respond to a series of needs of the group most affected by chronic conditions and situations of dependence [14,16].

Despite the existence of an integrated health and social system, there are shortcomings in relation to the diversity of cases for which the system is unable to respond. These include informal care and the burden on family members [9,12,17]; the impact on the quality of life of patients with chronic diseases [2,5]; the differential impact of chronic conditions according to sex and gender [18,19]; barriers to access to health or social services [20,21]; and, finally, a lack of coordination within the network of actors involved who are sometimes unaware of the extent to which all the needs are being met [20].

The population of the Bages region (Central Catalonia) shows a sustained increase in population aging and overaging [22], which favors a high prevalence of chronic diseases. Although chronic conditions are present in various stages of life, they become more complex to manage as age advances and there are more cases of multimorbidity and dependence. It is therefore crucial to have data and information on the needs of people affected by these conditions, their environment, and the services they require.

The Bages region, primarily a rural area, spans a surface of 1092 km^2^ and is characterized by the presence of only 2 urban centers [23]. As of 2021, the total population of the region was 179,770 [22]. Situated within Central Catalonia, the Bages region is 1 of the 7 regions that form the Catalan health system. The Catalan Health Institute (ICS) serves as the main health care provider in the area, covering approximately 85% of the population of Bages [24].

According to data from the Central Catalonia Territorial Management of the ICS [25], the prevalence of various chronic diseases grew between 2018 and 2021 (ICS, unpublished data, 2023). The most prevalent diseases were hypertension (40.2%), chronic pulmonary diseases (26.3%), and type 2 diabetes (16.1%). At the same time, the most prevalent mental disorders were anxiety (42.6%) and depression (18.3%), which also corresponded with the continued increase in chronic conditions and as one of the most common comorbidities. At the same time, the data showed differences by age and sex in the burden of chronic conditions, which reinforces the need to differentiate the approach by taking into account a sex and gender perspective [18,26-28].

Despite the availability of this information, mainly of a clinical nature, the ICS system does not have information on the needs of people with chronic and dependent conditions. It should be considered that the Bages area has a structure of health and social health services and providers included in the public system other than the ICS [14]. At the same time, social services depend on local public administration, which is governed by different legislation from the health sector [29]. Therefore, there is a lack of a procedure to bring together all these institutions and actors that provide care to people with chronic conditions and dependence across the board.

Given these conditions, in 2019, the Territorial Specialization and Competitiveness Project-Big Data, Analytics, Management and Strategy in Health and Social (Proyecto de Especialización y Competitividad Territorial – Big Data, Analytics, Gestión y Estrategia en Salud y Social [PECT BAGESS]) project was born. It was formed by several agents of the health and social system, public administration, technological sector, and university and research sector in the Bages region. The objective of the project is to address the problems and needs arising from dependence and chronic conditions and their impact on the health and social spheres, developing solutions, products, and services that can also become opportunities for economic growth.

This protocol presents one of the operations of the PECT BAGESS, which corresponds to the creation of a Laboratory for Statistics and Analysis of Dependence and Chronic Conditions in Bages. This will serve as the basis for coordinating information from all project entities. It will also make it possible to define the chronic conditions and conditions that generate dependence in the Bages area; identify their prevalence, incidence, and main associated factors; and propose products and services that can respond to these needs.

The PECT BAGESS project is currently being developed by 7 partners, with funding from the Generalitat de Catalunya [30], and 4 collaborating entities without funding. The coordination of the project is led by Manresa Town Hall.

## Methods

### Theoretical Approach

In order to address such a complex challenge as the one posed by the PECT BAGESS project, the Bages Shared Health and Social Agenda was drawn up. The Shared Agendas are initiatives promoted by different agents in the area with the aim of implementing a coordinated approach to complex challenges linked to the Sustainable Development Goals and the problems and opportunities arising from them [31].

The Bages Shared Agenda aims to bring together the relevant main actors in the area who will adopt a proactive approach to the challenge. In this way, objectives, resources, and structures can be shared, and collaborative and multidisciplinary solutions can be generated. In short, it is structured to satisfy the need for applying enabling technologies in this highly complex ecosystem relating to dependence and chronic conditions, placing the person living with these types of conditions and the needs arising from their health circumstances at the center. The main actors in the area who participate in the Shared Agenda are part of the health and social sector, both from the public administration, research centers, universities, and health care centers at all levels (eg, from primary care, specialized consultations, acute care, and chronic diseases).

The structure of the Shared Agenda, and consequently the PECT BAGESS project, in this environment of chronic conditions and dependence is framed within the strategy promoted by the Research and Innovation Smart Specialization Strategy (RIS3) of the Generalitat de Catalunya in the RIS3CAT 2030 edition. This commitment to transformative and responsible research and innovation is based on shared agendas as the main drivers to guide Catalonia toward a greener, digital, resilient, and fairer socioeconomic model [32]. In addition, the use of technology and a quadruple helix structure (4H) are promoted: patients and the population, health and social system, science and technology, and business. The joint work of these sectors and agents in the region will make it possible to provide a united response to the needs associated with the chronic conditions and population with dependencies in Bages, to export the dynamics and solutions generated to other regions of Catalonia once the project is completed.

The PECT BAGESS project seeks to improve the quality of life and promote the social inclusion of people with dependence and chronic diseases, making them active agents of their own health. Furthermore, it also wants to drive the economic transformation of the county with a focus on the digital transition of the health and social sector, both public and private, to make it a more competitive, sustainable, resilient, and quality service.

To this end, one of the operations to be carried out is the definition and detection of the needs of people in these circumstances. For this reason, mechanisms need to be established for sharing data and information among the different actors involved in the project, especially among those providing direct health care or population health care. The availability of real data on chronic conditions and dependence will make it possible to go deeper into the other aspects of the project, as well as to offer subsequent analyses and evaluations in greater depth.

As part of the study, data from supplier entities will be combined. In order to have information corresponding to a single person, the data will be merged using a unique identifier for any of the provider entities. This strategy ensures that the combined data are accurate in terms of ownership, although the problem in its implementation lies in the existence of a common unique key (Table 1).

**Table 1:** Characterization of the data sets involved in the combination process.

Characteristics	Health data	Social services data	Context data
Unique identifier	Personal identification code (PIC), personal ID	Personal ID	Personal ID
Additional information	Sex, age, municipality, nationality	Sex, age, municipality, nationality	Sex, age, municipality, nationality
Thematic data	Diagnosis	Problem, dependence	Income, living conditions

The utilization of a unique patient identifier is a complex yet essential aspect of the data merging process. Its purpose is to prevent duplication and ensure a comprehensive understanding of regional requirements and attributes. Recent literature delves into the discussion surrounding data sharing and provides insights into the implementation of unique patient identifiers [33-35], highlighting the complexities associated with integrating data, particularly personal data, from diverse sources. Furthermore, it is essential to address the legal implications before creating the database, as outlined in the “Ethical Considerations” section.

### Project Partners

The collaboration of various entities in the area, through research and implementation of comprehensive and innovative digital solutions, make the PECT BAGESS project possible. Among the partners (entities receiving funding) are Manresa Town Hall, as project coordinator; Sant Andreu Salut Fundació; Althaia Xarxa Assistencial de Manresa Fundació Privada; Fundació Eurecat; Institut Universitari d’Investigació en Atenció Primària-Institut Català de la Salut de la Catalunya Central (IDIAPJGol-ICS); Fundació Universitària del Bages-UManresa; and, finally, Fundació Ampans.

Also collaborating are the Regional Council of Bages, the Unió Consorci Formació, the Associació Catalana d’Entitats de Base Associativa, and the Fundació TIC Salut Social.

### Project Structure and Interconnection Between Operations

#### Overview

**Figure 1:**
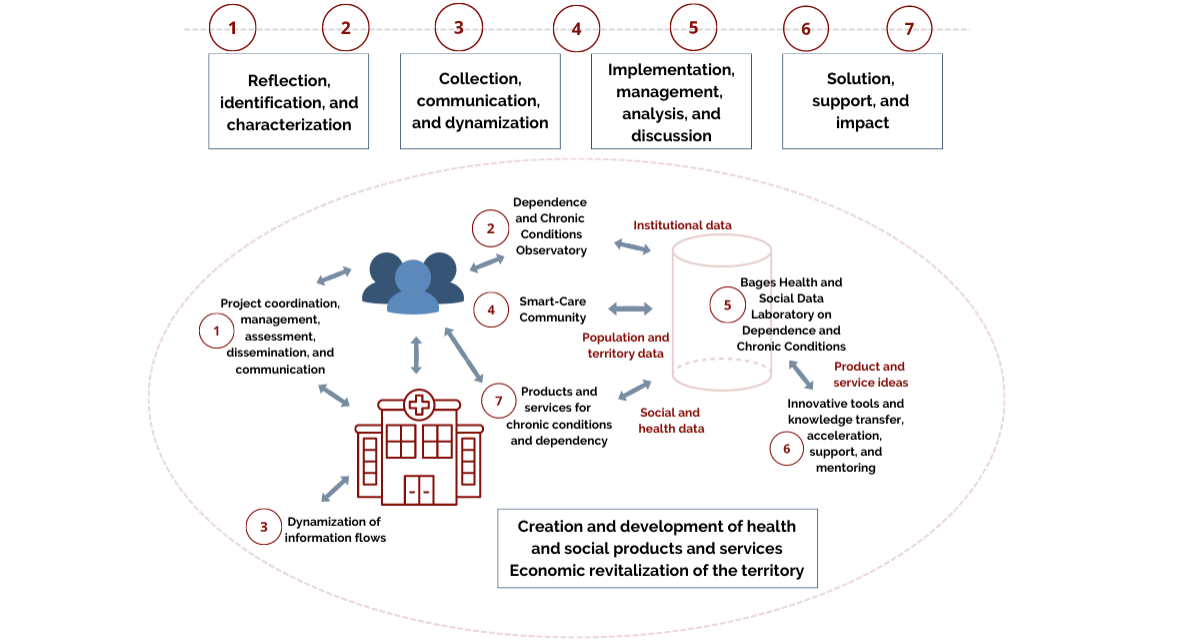
Structure of the Proyecto de Especialización y Competitividad Territorial-Big Data, Analytics, Gestión y Estrategia en Salud y Social (PECT BAGESS) and interconnection of operations.

The various entities involved in the project are key players in the 4 phases defined by the PECT BAGESS: (1) reflection, identification, and characterization; (2) collection, communication, and dynamization; (3) implementation, management, analysis, and discussion; and (4) solution, monitoring, and impact. These 4 phases group 7 operations, which are determined by the performance of each partner entity in each of them (see Figure 1).

#### Operation 1: Coordination, Management, Evaluation, Dissemination, and Communication of the PECT BAGESS

Manresa Town Hall is leading the transverse coordination operation. This allows one to have an overall view of the rest of the operations, facilitate their management, and ensure that the project is on schedule. It also supports each individual entity in terms of administrative and economic justification, while acting as a liaison between the entities and the General Directorate of Local Administration.

#### Operation 2: Observatory of Dependence and Chronic Conditions in Bages

Developed by Sant Andreu Salut, the objective of this operation is to manage the information generated about chronic conditions and dependence, both for the affected person and his or her family and carer. The observatory aims to be a tool for sharing needs and resources for this group, as well as gathering new actions and relevant information in this regard.

#### Operation 3: Dynamization of Information Flows

Althaia, responsible for the management and dynamization of information flows, seeks to incorporate the use of technologies to contribute to the transformation of the health care model, promoting a better patient-professional relationship and improved coordination among professionals. Using support tools, shared among the different health and social care providers, the aim is to contribute to better patient stratification and predictive medicine, thus minimizing acute cases and, consequently, regulating the need for health care resources. In this way, the quality of life of patients with chronic diseases is improved, and their care is made more efficient.

#### Operation 4: Smart-Care Community

Eurecat develops information and communication technology (ICT) tools for the integrated care of patients with chronic diseases, in a global context that takes into account the patient, caregivers, and health care environment. The center uses technologies based on artificial intelligence and human-machine interaction interfaces. This operation involves co-designing, implementing, and validating various management, learning, and support tools for the patient-professional care and social system. Eurecat also conducts 2 pilot tests in cardiac rehabilitation and mental health (schizophrenia), which are implemented in coordination with agents using these tools.

#### Operation 5: Statistical and Intelligent Analysis Laboratory

The collection of chronic condition and dependence data and their analysis are carried out at IDIAP JGol-ICS Catalunya Central through the Laboratory of Statistics and Intelligent Analysis. With the legal requirements to do so, with the completion of a legal study and an impact assessment, this operation makes it possible to gather all the existing data in terms of health and social data to detect the problems and needs of people with chronic conditions and dependence and their environment. Similarly, this operation serves to validate the interventions proposed by health care institutions, as well as the products and services of companies in the sector, and to propose new solutions based on the scientific evidence identified by the analysis.

#### Operation 6: Transformative and Knowledge Transfer, Acceleration, Support, and Monitoring Tools

The Fundació Universitària del Bages-UManresa focuses on the creation of a platform for entrepreneurship and support for health and social initiatives (eHealthinking) in the process of developing products and services in this sector, providing expertise in this type of process from a position of knowledge of existing structures. Both initial financing (Business Angels network) and support from the business network with expertise in the health-social field (Health and Social Innovation Cluster) will be provided.

#### Operation 7: Dependence Products and Services

The latest operation is led by Fundació Ampans, with the aim of creating a product that offers people with chronic conditions and dependence and in need of support solutions and tools that empower and facilitate the management of their daily reality. In the form of a technological tool (mobile app and back end platform), this product will enable the concerned person to find specialized professional social and health care services for their condition. Additionally, it will provide them with access to a library of resources, tools, and knowledge from collective experiences and enhance social relationships by finding specific recreational activities. This technological solution seeks to build change, fostering empowerment, quality of life, and person-centered care and focusing on meeting the needs of the dependent population.

### Creation of a Laboratory for Statistics and Analysis of Dependence and Chronic Conditions in Bages

In light of the project’s overarching framework outlined previously, we will now proceed to describe the implementation of the operation assigned to IDIAPJGol-ICS, which corresponds to operation 5. This consists of the following 2 elements: (1) description of the procedures associated with the laboratory and (2) description of the implementation proposal to be carried out.

#### Procedures Associated With the Laboratory

The procedures for operation of the laboratory are as described in the following paragraphs.

**Figure 2:**
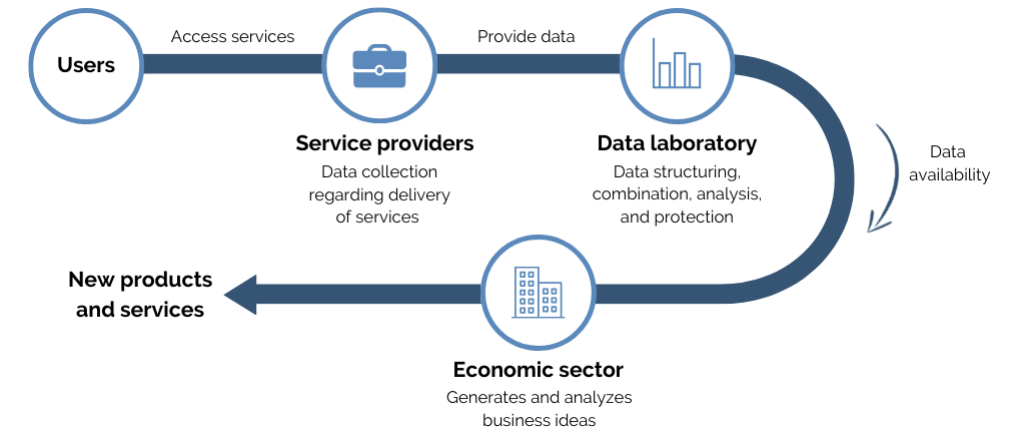
Procedure 1: collection, combination, and publication of chronic condition and dependence data.

First, data collection in the health, social, and contextual areas covers the data life cycle from its contribution by users at the time of access to services including the generation of new services in the area of chronic conditions and dependence by the economic fabric (Figure 2).

**Figure 3:**
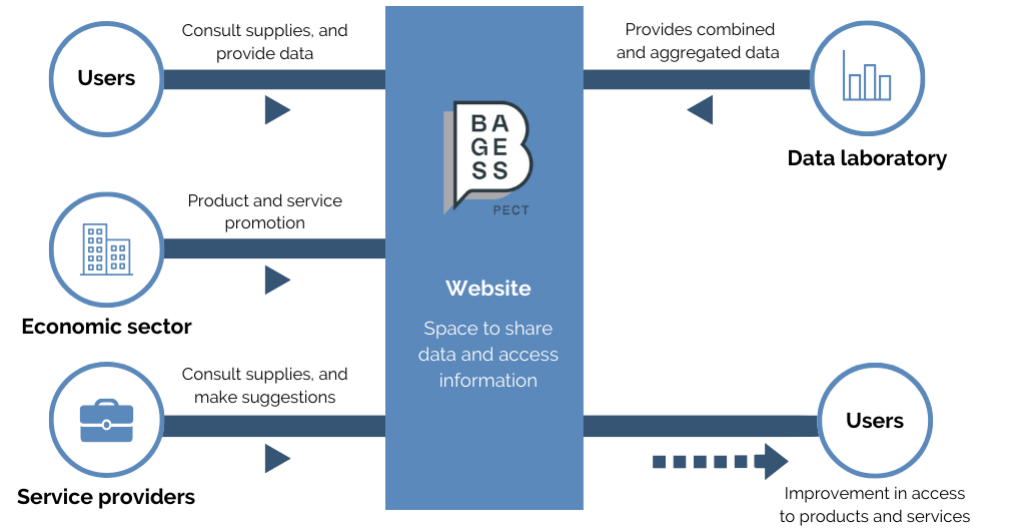
Procedure 2: transfer of data to contribute to the implementation of operations 4 and 6 of the Proyecto de Especialización y Competitividad Territorial-Big Data, Analytics, Gestión y Estrategia en Salud y Social (PECT BAGESS) project.

Second, data that contribute to performing operations 4 and 6 of the PECT BAGESS project are transferred. One of the first uses of the data obtained by the statistics laboratory is to deliver data to the ICT tools developed in the context of operations 4 and 6 of the PECT BAGESS project (Figure 3).

#### Description of the Implementation Proposal

In accordance with the previous paragraphs, the proposal involves creating the chronic condition and dependence laboratory with the implementation characteristics described in the following paragraphs.

First, the combination of the data will be carried out by combining 2 strategies: (1) use of pseudonymized data in which a unique identifier (personal ID) is used as the basis for the generation of the pseudonym combined with the person’s date of birth for the combination of health and social data and (2) use of general demographic data for the combination of contextual data.

Second, IDIAPJGol separates the functions in its project according to the following 2 blocks: (1) technical block, which is part of the process of pseudonymization, combination of data, creation of the data repository, obfuscation, and aggregation and preparation of data for dissemination to third parties, and (2) research block, which involves data analysis tasks and review of scientific evidence, needs assessment, and proposal of products and services.

Third, the pseudonymization process is carried out in a distributed manner both in the generation of the pseudonym and in the custody of the translation dictionaries and consists of the following entities: (1) pseudonymization neutral point that is in charge of obtaining the hash by applying the algorithms (SHA256 hash with a fixed salt not specified in the database and a pepper of 3 characters in length) and of keeping the resulting pseudonym values and of the entity making the request and (2) provider entities that are in charge of keeping the translation dictionary of the entity’s own pseudonyms.

Going into more detail on the operation, it is necessary to describe the agents involved in the process and their main function in relation to the process (except for the service users, who are not considered even though they are the ones providing the data, since they do not perform interactions at the information system level), which are as follows:

- Entity providing health or social services, which provides the data and keeps the pseudonym dictionaries of its own users
- Pseudonymization neutral point (IDIAPJGol-technical), which performs the pseudonymization process and the keys to obtain it
- Statistical laboratory (IDIAPJGol-technical), which prepares and keeps the pseudonymized data
- Research team (IDIAPJGol-research), which analyzes the pseudonymized data
- Economic fabric, which is the recipient of the aggregated data containing specially protected data but in which the individual is not identifiable

**Figure 4:**
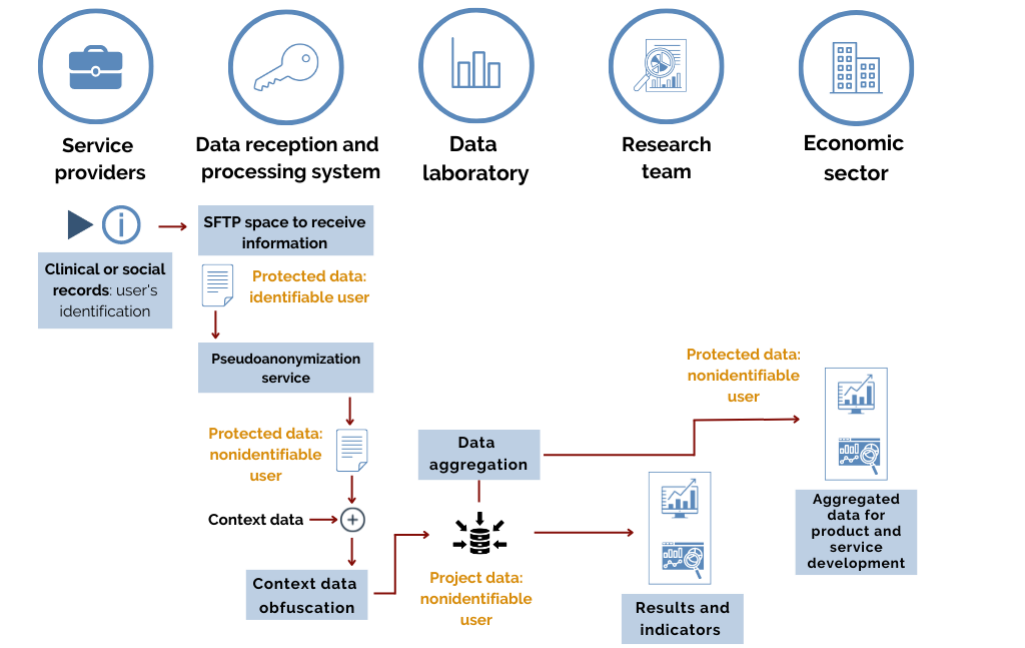
Global model proposed by the creation of the laboratory. SFTP: secure file transfer protocol.

The overall model that would emerge from this description can be seen in Figure 4.

### Monitoring and Assessment

Monitoring will be done in cooperation with the various partners involved in the project. The various operations will be evaluated by each entity, through process and results indicators, in order to meet the project’s objectives. Quality measures of the activities carried out will also be provided, assessing whether the objectives are being met and their impacts, always taking into account new needs that may arise throughout the process. The evaluation will be shared with all the participants regularly involved.

### Communication and Dissemination

The results of the project will be disseminated through the following different strategies led by the various entities participating in the project:

- PECT BAGESS website and social networks
- Observatory of Dependence and Chronic conditions (operation 2)
- Citizen science meetings and events
- Elaboration of a Policy Brief that includes recommendations for better care and prevention of the needs derived from chronic conditions and dependence, based on the experience of Bages
- Reports, presentations, and meetings with policy makers, health and social professionals, and other relevant stakeholders and organizations
- Scientific publications
- Presentations at local and national conferences

### Ethical Considerations

This project has been reviewed and approved by the Ethics Committee of IDIAPJGol (22/068-P). All activities carried out within the framework of this study will be conducted in accordance with existing ethical guidance, as indicated in the Universal Declaration on Bioethics and Human Rights adopted by UNESCO (19/10/2005); the Council of Europe Convention for the Protection of Human Rights and Dignity of the Human Being with regard to the Application of Biology and Medicine (1997) and its Additional Protocol on Biomedical Research (2005); the Declaration of Helsinki (2013) and relevant European Union laws (Directive 2001/20/EC of the European Parliament and of the Council); the Spanish Law on Biomedical Research (14/2007); and the Spanish Law on Personal Data Protection (3/2018). This analysis was also carried out considering the legislative aspects in the field of health and social services provision, as well as those related to the protection of personal data.

With regard to general ethical aspects, the following have been considered: social and scientific value of research, equitable search in relation to the resources of the population, use of widespread informed consent, processing of data from vulnerable groups.

Regarding the social and scientific value of research, the aim of the study is to obtain combined data on chronic conditions and dependence of the population of Bages. From the data obtained, it will be possible to better characterize the needs of the population (regardless of their characteristics), and as a result of the new products and services that can be defined by the economic fabric, the health and well-being of people will be improved.

Regarding the equitable search in relation to the resources of the population, the result of the research should make it possible to cover the analysis of the global population regardless of its context and based on the propensity to need access to products and services linked to social and health services.

Regarding the use of widespread informed consent, in this phase of the research and under current data protection regulations, consent of the interested parties in the data processed is not obtained for its implementation.

Regarding the processing of data from vulnerable groups, the aim of the research is focused on the group of users benefiting from social and health services who, according to the definition of the project itself, are or may be in a situation of vulnerability. In this respect, the research is aimed at achieving new services to reduce the effects and, if possible, the presence of these situations of vulnerability.

In any case, one of the most relevant aspects is the one related to the processing of service users’ personal data, which are combined to obtain the statistics laboratory. These specially protected data are subject to the provisions of Law 21/2000 on the rights to information concerning health and patient autonomy and clinical documentation, the basic Law 41/2002 regulating patient autonomy and rights and obligations regarding information and clinical documentation, Law 12/2007 on social services, Eropean Union Regulation 2016/679 General Data Protection Regulation, and Organic Law 3/2018 on the protection of personal data and guarantee of digital rights, General Health Law 14/1986.

To ensure that the activities to be carried out in the project are conducted in accordance with the law, prior consultation was held with the Catalan Data Protection Agency. The authority’s response indicated that the project was covered by current data protection regulations, provided that it was part of a research project and worked with anonymized data, guaranteeing technical and functional separation of pseudoanonymization and the conduct of the research. Considering all the aforementioned information, the data will be stored in an ICS server, and the IDIAPJGol research team will be responsible for data processing.

## Results

With project start-up in 2021, all PECT BAGESS operations have been initiated and are in progress. As of June 2023, the operation corresponding to the creation of the Laboratory for Statistics and Analysis of Dependence and Chronic Conditions reached the legal study stage, which covers the suitability of use and combination of the data necessary to create the database.

Since the laboratory will primarily consist of personal data provided by various entities offering health and social services in the region, ensuring data protection is of paramount importance. Considering that processing personal data involves the use of special data categories and entails high risk, a data protection impact assessment (DPIA) was carried out following the WP248 guide of Article 29 of the Working Party of the European Data Protection Board.

IDIAPJGol carried out this DPIA through data protection, analyzing the typology of the data included in the new treatment, collection, integration, and data processing procedures, as well as technical and organizational measures applied, in order to minimize the risk associated with their treatment. As a result of the implementation of the planned measures, the final impact of the data processing involves a low level of risk, and therefore, no additional measures are necessary. Thus, the data processing resulting from the creation of the laboratory was deemed feasible from the point of view of personal data protection.

At the same time, coordination with the rest of the operations includes monthly meetings between entities and the organization of various communication events open to the authorities and the public of the Bages region, which have made it possible to publicize the work being done within the framework of this project.

## Discussion

This paper presents the project for the creation of the Laboratory for Statistics and Analysis of Dependence and Chronic Conditions in the Bages region, as part of the PECT BAGESS. As an ongoing project, the authors anticipate an improved data sharing process among partners that will be key to move toward a better understanding of the chronic conditions and dependent needs of the population in this area. This project should also provide a foundation for the scalability of this model in other regions.

**Figure 5:**
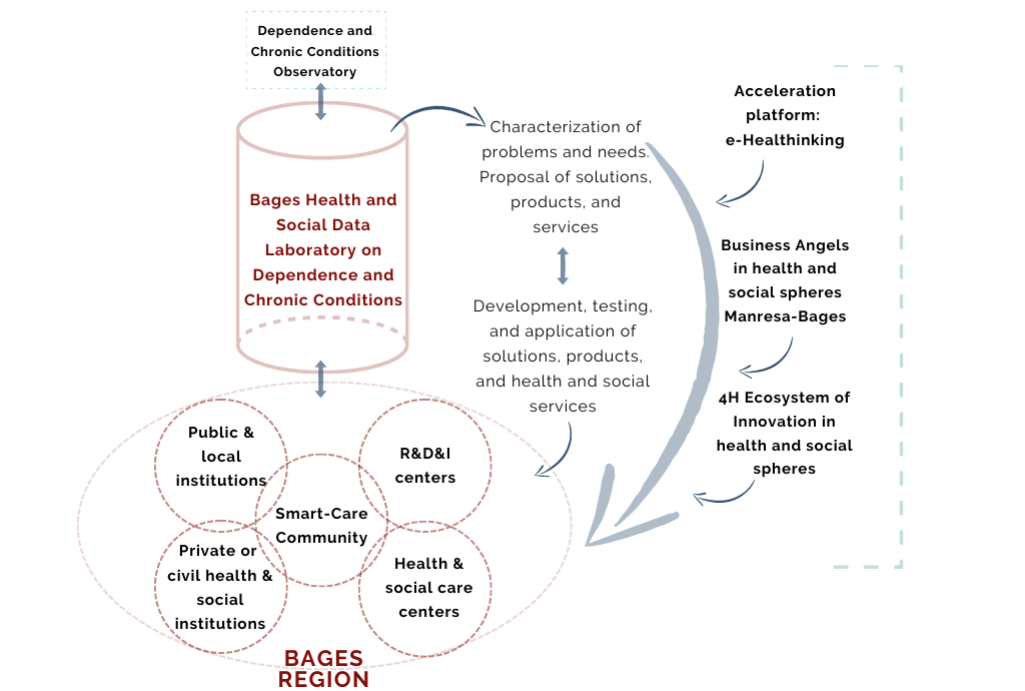
Relationship schemes, information flows, and management of the Big Data, Analytics, Gestión y Estrategia en Salud y Social (BAGESS) project, once the Proyecto de Especialización y Competitividad Territorial (PECT) has been passed. 4H: quadruple helix structure; R&D&I: research and design and innovation.

The authors and agents involved in this project expect that the resulting organizational dynamics and structures will allow for a model of health and social management that may have a remarkable impact on these sectors. Products such as the Observatory of Dependence and Chronic Conditions, the “eHealthinking” entrepreneurship platform, the Bages-Catalonia Central Business Angels organization, the Smart-Care Community citizen participation platform, or the Health and Social Innovation Cluster, as well as the websites and applications to be developed may be very useful for generating synergies and facilitating the living conditions of people who can benefit from all these services. In relational terms, the project seeks to culminate in interaction and collaboration in other health and social projects, whether they have an impact on research, innovation, or economic development (Figure 5).

To facilitate these dynamics, the backbone of the BAGESS project lies in the Health and Social database, which is fed by the different communication strategies and the information available from each entity, both from health care and other organizations related to dependence and chronic conditions. From the analysis of data from the Laboratory of Statistics and Analysis, it will be possible to extract the information that the Observatory of Dependence and Chronic conditions will collect to offer a more precise characterization of problems and needs related to dependence. As a result of these analyses, it will be possible to propose solutions, products, and services that will be shared with the BAGESS community, accompanied by research and innovation entities, and developed as business projects. These will contribute to the dynamization of the health and social structure and to the economic acceleration of the area. Previous experiences with similar projects can be found in southern Catalonia, where an integrated care model called Salut + Social was implemented. This model aimed to enhance coordination among professionals from health and social services, with a specific focus on addressing the needs of patients with chronic diseases and dependencies [36]. The program received positive acceptance among professionals and high satisfaction among users, improving the efficiency of care and preventing duplication of tasks [37]. Despite not covering the scope of the PECT BAGESS (which also includes economic and technologic agents), this experience confirms the benefits of joining forces to look after a population with potentially high health and social care needs.

We acknowledge, however, that this study presents several limitations. The potential risks of the implementation of this project will be detected at the time of implementing this management model, as well as the limitation of database maintenance, which will require resources beyond those that have been developed for this project, although several options are currently being considered and coordinated by the Generalitat de Catalunya. Moreover, the commitment and availability of the entities involved in taking on the current tasks and the possible continuation of the tools and products derived from the project must be taken into account, although, so far, the coordination and communication between them have been very positive. Finally, there is a risk arising from the nature of the information with which we work, since it refers to personal health and social data; therefore, it is very necessary to adhere to the corresponding regulations in order to maintain its confidentiality. Being aware of the latter, we developed a legal study within the framework of the operation presented in this protocol, in order to rely on the necessary legal support to develop the project.

In conclusion, the proposal discussed in this study emphasizes the importance of finding a balance between the needs of the population and the protection of data privacy. Implementing secure data-sharing models in health and social care systems can lead to improved efficiency and satisfaction among both professionals and the population. It also presents an opportunity to foster synergies and develop new products and services that cater to the specific needs of individuals with chronic diseases and dependencies. Additionally, the creation of comprehensive databases plays a crucial role in generating evidence and facilitating research in these areas of increasing prevalence. However, it is essential to adhere to frameworks and regulations that ensure the secure transmission and handling of data, whether at the local or international level. By establishing robust and protected circuits, the potential benefits of data sharing can be realized while maintaining the privacy and confidentiality of individuals’ personal information.

## Abbreviations

4H: quadruple helix structure
DPIA: data protection impact assessment
ICS: Institut Català de la Salut (Catalan Health Institute)
ICT: information and communications technology
IDIAPJGol: University Institute for Research in Primary Health Care Jordi Gol i Gurina
PECT BAGESS: Territorial Specialization and Competitiveness Project-Big Data, Analytics, Management and Strategy in Health and Social (Proyecto de Especialización y Competitividad Territorial-Big Data, Analytics, Gestión y Estrategia en Salud y Social)
RIS3: Research Innovation Strategies for Smart Specialization

## Data Availability

Access to the data used in this article is limited to the Catalan Health Institute due to the sensitive nature of personal data and the requirements set forth by Spanish and European regulations. As a result, the data cannot be openly shared. However, the results mentioned in the Introduction section will be presented in a forthcoming manuscript by Pujolar et al titled “A Gender-Based Approach to Chronic Conditions in Central Catalonia (Spain): A Descriptive Cross-Sectional Study” (in preparation as of 2023).

Furthermore, the creation of the laboratory outlined in this protocol aims to facilitate broader access to data within the established legal and ethical frameworks.

## References

[1] Reynolds R, Dennis S, Hasan I, Slewa J, Chen W, Tian D, Bobba S, Zwar N (2018). A systematic review of chronic disease management interventions in primary care. BMC Fam Pract.

[2] Schaink AK, Kuluski K, Lyons RF, Fortin M, Jadad AR, Upshur R, Wodchis WP (2012). A scoping review and thematic classification of patient complexity: offering a unifying framework. J Comorb.

[3] Hernansanz Iglesias F, Martori Cañas JC, Limón Ramírez E, Alavedra Celada C, Blay Pueyo C (2021). Clustering complex chronic patients: a cross-sectional community study from the general practitioner's perspective. Int J Integr Care.

[4] Limón E, Blay Pueyo C, Santaeugenia González S, Carles Segura J, Hernansanz Iglesias F, Alavedra Celada C (2017). Cronicitat i complexitat clínica. Butlletí de l'Atenció Primària de Catalunya.

[5] Poitras ME, Maltais ME, Bestard-Denommé L, Stewart M, Fortin M (2018). What are the effective elements in patient-centered and multimorbidity care? A scoping review. BMC Health Serv Res.

[6] Davy C, Bleasel J, Liu H, Tchan M, Ponniah S, Brown A (2015). Effectiveness of chronic care models: opportunities for improving healthcare practice and health outcomes: a systematic review. BMC Health Serv Res.

[7] (2018). Multimorbidity: a priority for global health research. The Academy of Medical Sciences.

[8] Betancourt MT, Roberts KC, Bennett T, Driscoll ER, Jayaraman G, Pelletier L (2014). Monitoring chronic diseases in Canada: the Chronic Disease Indicator Framework. Chronic Dis Inj Can.

[9] García-Calvente M, Mateo-Rodríguez I, Maroto-Navarro G (2004). El impacto de cuidar en la salud y la calidad de vida de las mujeres. Gac Sanit.

[10] Mármol-López MI, Miguel Montoya I, Montejano Lozoya R, Escribano Pérez A, Gea Caballero V, Ruiz Hontangas A (2018). Impacto de las intervenciones enfermeras en la atención a la cronicidad en España. Revisión sistemática. Revista Española de Salud Pública.

[11] García Calvente MDM, del Río Lozano M, Marcos Marcos J (2011). Desigualdades de género en el deterioro de la salud como consecuencia del cuidado informal en España. Gaceta sanitaria.

[12] Abajo M, Rodríguez-Sanz M, Malmusi D, Salvador M, Borrell C (2017). Gender and socio-economic inequalities in health and living conditions among co-resident informal caregivers: a nationwide survey in Spain. J Adv Nurs.

[13] Kogan AC, Wilber K, Mosqueda L (2016). Person-centered care for older adults with chronic conditions and functional impairment: a systematic literature review. J Am Geriatr Soc.

[14] (2018). El CatSalut i el model sanitari català. Servei Català de la Salut.

[15] Baker R, Freeman GK, Haggerty JL, Bankart MJ, Nockels KH (2020). Primary medical care continuity and patient mortality: a systematic review. Br J Gen Pract.

[16] Generalitat de Catalunya (2016). L'atenció centrada en la persona en el model d'atenció integrada social i sanitària de Catalunya. Pla interdepartamental d'atenció i interacció social i sanitària.

[17] Villanueva Lumbreras A, García-Orellán R (2018). Calidad de vida del cuidador informal: un análisis de concepto. Rev Ene Enfermería.

[18] Criado Perez C (2019). Invisible women: exposing data bias in a world designed for men.

[19] Malmusi D, Artazcoz L, Benach J, Borrell C (2012). Perception or real illness? How chronic conditions contribute to gender inequalities in self-rated health. Eur J Public Health.

[20] Fernández-Medina IM, Ruíz-Fernández MD, Gálvez-Ramírez F, Martínez-Mengíbar E, Ruíz-García ME, Jiménez-Lasserrotte MDM, Ortega-Galán ÁM, Hernández-Padilla JM (2021). The experiences of home care nurses in regard to the care of vulnerable populations: a qualitative study. Healthcare.

[21] Duran Heras MÁ (2017). Los costes no sanitarios de la atención a los pacientes crónicos. https://digital.csic.es/handle/10261/147902.

[22] Institut d'Estadística de Catalunya (2022). Població a 1 de gener. Comarques i Aran, àmbits i províncies.

[23] Vidal-Alaball J, Mendioroz Peña J, Sauch Valmaña G (2018). Rural-urban differences in the pattern of referrals to an asynchronous teledermatology service. International Archives of Medicine.

[24] (2021). Primary Care Services Information Systems, Gerència Territorial de la Catalunya Central. Institut Català de la Salut.

[25] (2022). L'ICS. Institut Català de la Salut.

[26] García Calvente MDM, Jiménez Rodrigo ML, Martínez Morante E (2010). Guía para incorporar la perspectiva de género en la investigación en salud.

[27] Valls-Llobet C (2001). Desigualdades de género en Salud Pública. Quadern CAPS.

[28] Sen G, Östlin P, George A (2007). Unequal, Unfair, Ineffective and Inefficient Gender Inequity in Health: Why it exists and how we can change it?. Women and Gender Equity Knowledge Network (WGEKN).

[29] (2022). El Sistema català de serveis socials. Departament de Drets Socials.

[30] Departament de la Presidència (2020). ORDRE PRE/197/2020, de 10 de novembre, de modificació de l'Ordre PRE/161/2019, d'1 d'agost. Diari Oficial de la Generalitat de Catalunya.

[31] Fernández T, Romagosa M (2020). L'articulació d'agendes compartides per a la sostenibilitat i el canvi social. Una contribució des del territori al debat de la UE sobre les transicions cap a la sostenibilitat. Monitoratge de la RIS3CAT.

[32] (2022). RIS3CAT 2030: Estratègia per a l'especialització intel·ligent de Catalunya 2030. Generalitat de Catalunya.

[33] Ranchon F, Chanoine S, Lambert-Lacroix S, Bosson JL, Moreau-Gaudry A, Bedouch P (2023). Development of indirect health data linkage on health product use and care trajectories in France: systematic review. J Med Internet Res.

[34] Kalkman S, Mostert M, Gerlinger C, van Delden JJM, van Thiel GJMW (2019). Responsible data sharing in international health research: a systematic review of principles and norms. BMC Med Ethics.

[35] Nurmi SM, Kangasniemi M, Halkoaho A, Pietilä AM (2019). Privacy of clinical research subjects: an integrative literature review. J Empir Res Hum Res Ethics.

[36] Gavaldà-Espelta E, Lleixà-Fortuño MDM, Baucells-Lluis J, Ferré-Ferraté M, Mora-López G, Tomàs-Navarro B, Curto-Romeu C, Lucas-Noll J, Aguilar Martin C, Gonçalves AQ, Ferré-Grau C (2020). Effectiveness of the integrated care model Salut+Social in patients with chronic conditions: A mixed methods study protocol. Medicine (Baltimore).

[37] Gavaldà-Espelta E, Lleixà-Fortuño MDM, Aguilar Martín C, Pozo M, Ferré-Ferraté M, Tomàs-Navarro B, Curto-Romeu C, Lucas-Noll J, Baucells-Lluis J, Gonçalves AQ, Ferré-Grau C (2022). Integrated care model assessment by professionals, informal caregivers and chronic or social dependent patients: a qualitative study. Int J Environ Res Public Health.

